# Does femoral tunnel location have an effect on functional and clinical results in medial patellofemoral ligament reconstruction?

**DOI:** 10.55730/1300-0144.5965

**Published:** 2024-11-22

**Authors:** İbrahim KAYA, Murat ÇİÇEKLİDAĞ, Resul BİRCAN, Mustafa ODLUYURT, Abdurrahman VURAL, İnci Hazal AYAS, Muhammet Baybars ATAOĞLU, Ulunay KANATLI

**Affiliations:** 1 Department of Orthopedics and Traumatology, Abdurrahman Yurtaslan Oncology Training and Research Hospital, Ankara, Turkiye; 2 Department of Orthopedics and Traumatology, Faculty of Medicine, Ankara Yıldırım Beyazıt University Yenimahalle Training and Research Hospital, Ankara, Turkiye; 3 Department of Orthopedics and Traumatology, Çaycuma State Hospital, Zonguldak, Turkiye; 4 Department of Orthopedics and Traumatology, Başaksehir Cam and Sakura City Hospital, İstanbul, Turkiye; 5 Department of Physiotherapy and Rehabilitation, Faculty of Health Sciences, Gazi University, Ankara, Turkiye; 6 Department of Orthopedics and Traumatology, Faculty of Medicine, Gazi University, Ankara, Turkiye

**Keywords:** Medial patellofemoral ligament reconstruction, patellofemoral instability, femoral tunnel, Schottle’s point, functional outcome

## Abstract

**Background/aim:**

Multiple reconstruction techniques have been described to mimic the normal anatomy and physiology of medial patellofemoral ligament (MPFL) reconstruction. The success of MPFL reconstruction depends on various factors such as graft selection, the location of the patellar tunnel and femoral tunnel (FT), and graft fixation methods. The aim of this study was to analyze the relationship between FT location and functional and clinical outcomes after MPFL reconstruction.

**Materials and methods:**

The midterm clinical results of patients who underwent MPFL reconstruction for patellofemoral instability in a single institution between 2013 and 2019 were evaluated retrospectively. If the FT was within the 6-mm-diameter reference circle, the center of which is Schottle’s point, the tunnel was accepted as anatomical; otherwise, it was considered a nonanatomical tunnel. The functional outcomes of the patients in both groups were evaluated with Kujala, Tampa kinesiophobia, and visual analog scale (VAS) pain scoring.

**Results:**

A total of 34 patients, 23 female (67.6%) and 11 male (32.4%), were evaluated. The mean follow-up period was 48.92 ± 2.93 (months). Tunnel position was anatomical in 22 patients (64.7%) and nonanatomical in 12 (35.3%). The postoperative VAS pain scores of those in the anatomical tunnel group were significantly lower than those in the nonanatomical tunnel group (p = 0.015). There was no statistically significant difference between the groups in terms of Kujala or Tampa kinesiophobia scores (p > 0.05).

**Conclusion:**

Although FT placement did not affect functional scores in MPFL reconstruction in this study, malpositioning of the FT is associated with a higher postoperative VAS pain score.

## 1. Introduction

Patellofemoral instability (PFI) can be defined as abnormal, symptomatic, medial–lateral displacement of the patella within the trochlear groove [1]. The probability of redislocation after the first occurrence of patellar dislocation is 15%–44% [1,2], while it is higher than 50% after the second [3,4]. Surgical management is recommended in patients with recurrent patellar dislocation due to high recurrence rates with conservative management [4]. The medial patellofemoral ligament (MPFL) is the most important passive stabilizing structure against lateral patellar translation. It restricts lateral translation of the patella by 60% when the knee is performing flexion between 0° and 30°; thus it is a very important structure for patellofemoral stability [5,6]. Studies have shown that the MPFL injury that occurs after patellar dislocation is the most important factor causing recurrent patellar dislocation, and therefore MPFL reconstruction has become an accepted method to treat recurrent PFI [7–9].

While MPFL reconstruction can be performed in isolation, it can also be applied concurrently with bony procedures in patients with lower extremity malalignment, trochlear dysplasia, or increased tibial tubercle–trochlear groove (TT–TG) distance [10]. Multiple reconstruction techniques have been described to mimic the normal anatomy and physiology of the MPFL [1,11,12]. There was no standardized reconstruction protocol at the time of writing this article. The success of the reconstruction depends on various factors such as graft selection, the location of the patellar tunnel, the location of the femoral tunnel (FT), and graft fixation methods. Studies have been performed to investigate the effect of FT placement, and most surgeons agree that anatomical FT placement is essential for the success of treatment [13,14]. It has been shown that the more anterior and proximal placement of the FT compared to its normal anatomical position causes an increase in patellofemoral joint (PFJ) contact pressure at low flexion angles [14,15]. As the importance of FT position has been realized, many researchers have focused on defining the anatomically and radiologically appropriate FT placement, with the most popular and renowned one being defined by Schottle et al. [16].

The effect of FT location on clinical outcomes is still a controversial topic in the literature. The aim in the present study was to analyze the relationship between FT location and functional and clinical outcomes after MPFL reconstruction. The hypothesis of the study is that nonanatomical FT placement according to Schottle’s point will be associated with worse clinical and functional outcomes.

## 2. Materials and methods

The midterm clinical results of patients who underwent MPFL reconstruction for PFI in a single institution between 2013 and 2019 were evaluated retrospectively. The study protocol was approved by the local Ethics Committee (Date: XXXX, No: XXX). The study was conducted in accordance with the principles of the Declaration of Helsinki and written informed consent was obtained from all participants. Included in the study were patients that had MPFL rupture or laxity signs on magnetic resonance imaging (MRI) scans and that underwent MPFL reconstruction surgery for recurrent dislocations or failure to respond to conservative management after the first dislocation, a true lateral X-ray of the knee postoperatively, and finally a follow-up period of at least 24 months. Excluded were patients who underwent tibial tubercle osteotomy due to TT–TG distance or patella alta; those with Dejour type 3 and type 4 trochlear dysplasia, multiple ligament injuries, open physis line, and history of surgery due to patellofemoral instability; and those whose clinical and radiological data were lacking. After these criteria were evaluated, 34 knees of 34 patients were included in the study for data analysis.

### 2.1. Surgical technique

All operations were performed by a senior surgeon with more than 10 years of experience in patella reconstructive surgery. MPFL reconstruction was performed for all patients with the technique described by Christiansen et al. while using ipsilateral semitendinosus or gracilis tendon as an autograft [17]. In this technique, two parallel patellar tunnels with a diameter of 4.5 mm and a blind FT with a diameter of 6 mm and a length of approximately 25–30 mm are created.

### 2.2. Postoperative care and rehabilitation

All patients underwent the same rehabilitation program postoperatively. A hinged knee brace that was set to allow only 0°–30° of flexion was applied to the patients. Weight bearing was allowed as tolerated with crutches. Straight leg raise and isometric quadriceps exercises were started on the first postoperative day. The permitted flexion angle of the adjustable knee brace was increased by 30° every 2 weeks, and it was terminated in week 6. After week 8, strengthening exercises were started. After 6 months postoperatively, when the patient achieved full range of motion and strength, full return to sports was allowed.

### 2.3. Clinical and radiological evaluation

The patients preoperative visual analog scale (VAS) pain score, total number of patellar dislocations, and time from the first patellar dislocation to surgery were recorded. Postoperative patients were reevaluated at their final follow-up, which was a minimum of 2 years after the surgery. Clinical and functional outcomes were evaluated using the Kujala score, the Tampa kinesiophobia scale, and the VAS score. Postsurgical complications of MPFL reconstruction such as patellar fracture, recurrent dislocation, and arthritis in the PFJ were also questioned and examined. Furthermore, quadriceps atrophy, knee range of motion, patellar apprehension sign, and patellar shift test were evaluated.

**Figure 1:**
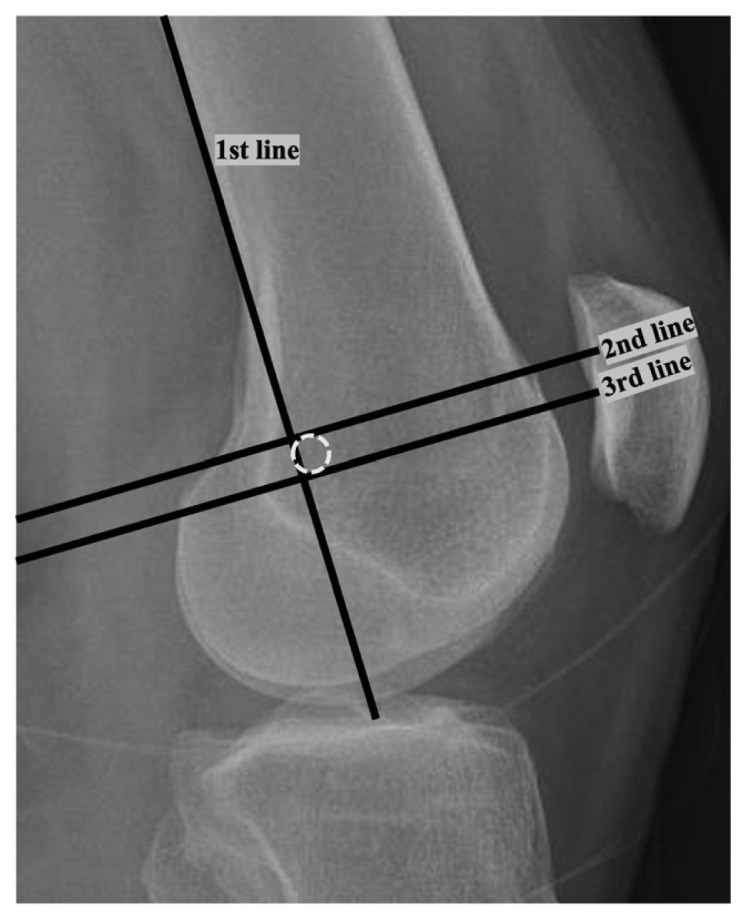
The distal extension of the posterior femoral cortex (1st line), the line drawn at a right angle from the intersection of the posterior of the medial condyle with the posterior femoral cortex to the 1st line (2nd line), and the line drawn at a right angle to the 1st line from the most posterior point of the Blumensaat line (3rd line).

**Figure 2:**
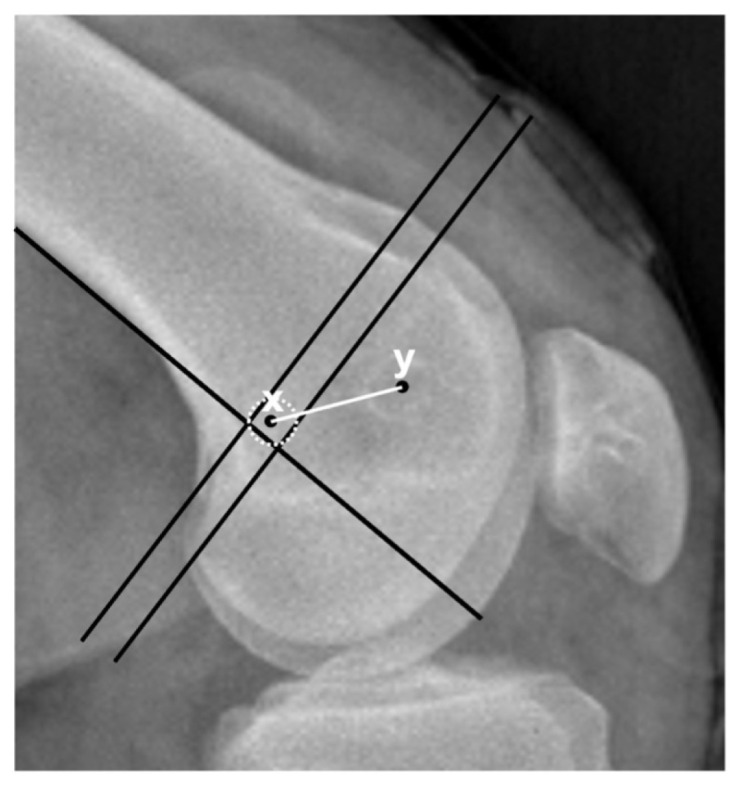
The distance between Schottle’s point (x) and the center of the femoral tunnel (y) was measured in millimeters and called the tunnel distance.

For radiological evaluation, routine postoperative anteroposterior knee and lateral knee X-rays at 30° flexion were used. The lateral X-rays were considered accurate if both condyles overlapped perfectly. To determine Schottle’s point using an accurate lateral knee X-ray, the distal extension of the posterior femoral cortex (1st line), the line drawn at a right angle from the intersection of the posterior of the medial condyle with the posterior femoral cortex to the 1st line (2nd line), and finally the line drawn at a right angle to the 1st line from the most posterior point of the Blumensaat line (3rd line) have to be determined. Using these lines, Schottle et al. defined a 5-mm circle with its center 1.3 mm anterior to the 1st line and 2.5 mm distal to the 2nd line [16] (Figure 1). Although Schottle defined all points of the MPFL insertion within a 5-mm-diameter circle, the reference anatomical circle diameter was accepted as 6 mm for evaluation, since a 6-mm-diameter FT was used in the surgical technique. The location of the FT opened for reconstruction was determined on the lateral knee radiography, and the distance to the Schottle point was measured and recorded as the tunnel distance (Figure 2). The evaluation of radiographs and measurements of tunnel localizations were performed by two authors of the present study. The measurement results of both observers were averaged to determine the exact measurements. The interobserver and intraobserver correlations of the measurements and evaluations were calculated. If the center of the FT was inside the 6-mm-diameter reference circle, the tunnel was accepted as anatomical; otherwise, it was considered a nonanatomical tunnel. The patients were divided into two groups according to whether their FTs were anatomical or nonanatomical. The functional outcomes of the patients in both groups were evaluated with Kujala, Tampa kinesiophobia, and VAS pain scoring.

### 2.4. Statistical analysis

SPSS (IBM SPSS Statistics 26) was used in the analysis and interpretation of the findings. The independent samples t-test (t-table value) statistics were used to compare the measurement values of two independent groups with a normal distribution. The Mann–Whitney U test (Z-table value) was used for comparison of measurement values of two independent groups that did not have a normal distribution; Wilcoxon’s test (Z-table value) was used to compare two dependent groups. The Fisher’s exact crosstabs were used according to the expected value levels in examining the relations of two qualitative variables with each other. The sample size of our study was calculated by statistical analysis based on the data of a similar study by Hopper et al. [18]. The difference between the two groups was calculated with a sample size of 5% alpha level and 80% power. The minimum number of patients was found to be 17; 11 for the anatomical tunnel group and 6 for the nonanatomical tunnel group.

## 3. Results

A total of 34 patients, 23 female (67.6%) and 11 male (32.4%), were evaluated in the study. Of the 34 knees, 13 (38.2%) were right knees and 21 (61.8%) were left knees. The mean age of all patients at the time of surgery was 23.57 ± 9.49 (years), the median was 23.6 (15.0–41.0) (years) (Table 1), and the mean follow-up period was 48.92 ± 2.93 (months), with a median of 38.3 (25.1–85.1) (months).

No recurrence or arthritis in the PFJ was observed in any of the patients during the follow-up. Postoperatively, tourniquet paralysis developed in 1 patient, and patella fracture developed in another patient, which was treated surgically. At the last postoperative follow-up, the range of motion of all patients was within the normal range. A positive patellar apprehension test result was still observed in 2 patients and quadriceps atrophy was observed in 1 patient. Tunnel position was not associated with a positive apprehension sign.

**Figure 3:**
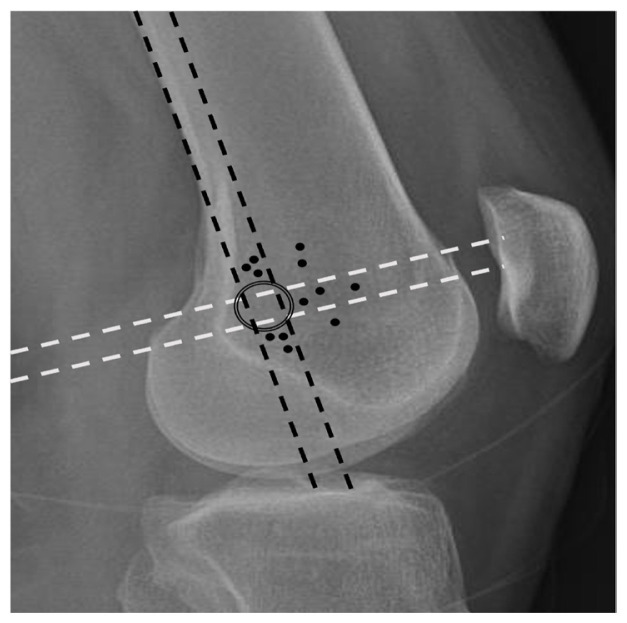
Distribution of nonanatomical tunnels.

It was determined that the tunnel position was anatomical in 22 patients (64.7%) and nonanatomical in 12 (35.3%). Further examination revealed that 41.7% of the nonanatomical tunnels were located in the superior or anterosuperior direction, 33.3% in the inferior or anteroinferior direction, and 25% in the anterior direction (Figure 3).

The minimum tunnel distance measured according to Schottle’s point of patients with an anatomical tunnel was 1.2 mm, while the highest distance was 2.8 mm and the mean was 2.14 ± 0.55 mm. In patients whose tunnel position is not anatomical, the shortest tunnel distance was 5.0 mm; the longest distance was 22.8 mm and the mean was 10.87 ± 4.94 mm (Table 1).

There was no statistically significant difference between the groups in terms of age (years) (p > 0.05). The tunnel position groups were independent of the age (year) variable and were homogeneous.

There was no statistically significant relationship between the anatomic/nonanatomical condition of the tunnel position and the operated side, sex, or complication status (p > 0.05). Anatomical/nonanatomical tunnel status was determined to be independent and homogeneous in terms of the specified features (Table 1).

There was no statistically significant difference between the groups in terms of preoperative VAS pain scores (p > 0.05). Postoperative VAS scores were significantly lower than preoperative scores in both groups (p < 0.05). A statistically significant difference was found between the groups in terms of postoperative VAS pain scores (Z = −2.443; p = 0.015). The postoperative VAS pain scores of those in the anatomical tunnel group were significantly lower than those in the nonanatomical tunnel group (Table 2).

There was no statistically significant difference between the groups in terms of Kujala or Tampa kinesiophobia scores (p > 0.05) (Table 3).

There was no statistically significant difference in the number of dislocations prior to surgery according to the anatomic/nonanatomical tunnel groups (p > 0.05). Total number of patellar dislocations did not have a statistically significant relationship with Kujala or Tampa kinesiophobia scores in either group (p > 0.05). While this was also true for the anatomic tunnel group for postoperative VAS scores, a positive, moderate, and statistically significant correlation was found between the number of dislocations and postoperative VAS pain scores for the patients with nonanatomical tunnels (r = 0.654; p = 0.015). Postoperative VAS pain scores increased as the number of dislocations increased in patients in the nonanatomical tunnel group. There was no statistically significant correlation for the time between trauma and surgery and Kujala, Tampa kinesiophobia, or postoperative VAS pain scores in either tunnel group (p > 0.05).

## 4. Discussion

The most important finding of the present study was that there was a statistically significant difference between the anatomic and nonanatomic tunnel groups in terms of postoperative VAS pain scores. This finding is an important contribution to the idea that anatomic FT position has beneficial effects on clinical results in MPFL reconstruction.

Many studies have been conducted to evaluate the effect of FT position in MPFL reconstruction and different results have been reported [18–20,23]. Hiemstra et al. [19] divided 155 knees on which they had performed MPFL reconstruction into three groups according to the distance between the center of the FT and Schottle’s point, as ideal (0–6 mm), good (>6–12 mm), and poor (>12 mm). They reported that no statistically significant relationship could be found between the accuracy of the tunnel location and the Banff Patellar Instability Instrument score. Similarly, Tscholl et al. [20] found that there was no significant difference in the Kujala scores of patients whose FT was inside or outside the accepted distance of 10 mm according to Schottle’s point and that the position of the FT did not have a significant effect on any subjective or objective clinical outcome, but they stated that a misplaced FT may lead to postoperative complications. Like those studies, it was reported that there was no correlation between FT location and functional and clinical outcomes in studies published by Servien et al. in 2010, McCarthy et al. in 2013, and Pandey et al. in 2018 [8,21,22]. Similarly, our study also showed no significant differences in Kujala or Tampa kinesiophobia scores between the two groups, thus showing that FT position does not affect functional scores.

There are also studies in the literature reporting a relationship between FT placement and postoperative clinical and functional outcomes. Larson et al. [23] stated that nonanatomical FT placement according to Schottle’s point causes decreased function and increased pain. Similarly, in the current study, patients with a nonanatomical FT location had more pain. Hopper et al. [18] performed a study in which 68 patients (72 knees) who underwent MPFL reconstruction were divided into two groups, namely anatomical (<10 mm) and nonanatomical (>10 mm), according to the distance between the center of the FT and Schottle’s point. In contrast to our study, it was reported that postoperative Kujala scores were significantly higher and fewer recurrent dislocations were reported in the anatomical tunnel group. This difference may have been due to the disparity in the anatomically accepted tunnel placement distance between the studies.

There are also conflicting biomechanical studies in the literature evaluating the effect of FT placement. Although there are studies [24] in which it is argued that nonisometric FT placement does not affect patellofemoral contact area and pressure, biomechanical studies by Stephen et al. [25] and Elias et al. [14] in 2016 showed that FT placement in the proximal and distal anatomical regions resulted in higher PFJ pressure during knee flexion and extension and caused nonphysiological patellar movement. They showed that correct positioning of the FT and graft tension are important in maintaining normal PFJ kinematics and articular cartilage contact stresses during MPFL reconstruction. There are studies reporting that incorrect placement in the proximal–distal direction while opening the FT creates more anisometry than incorrectly opening the tunnel in the anterior–posterior direction [26,27]. In the current study, 75% of the nonanatomical tunnels were placed in the aforementioned proximal–distal direction, which may have had an effect on the higher VAS scores of the nonanatomical tunnel group. The pressure increase seen in the PFJ after MPFL reconstruction can cause very dramatic complications such as arthritis in the long term. We think that the higher VAS pain scores of the patients in the nonanatomical tunnel group may have been related to the change in the graft isometry and the increase in PFJ contact pressures. Although there was no difference in functional scores between the two groups, we do not know whether PFJ degeneration will develop in patients in the long term. A longer follow-up may reveal an increased incidence of patellofemoral arthritis.

In the literature, different points referencing similar regions have been defined for FT placement, and the most popular one was defined by Schottle et al. [16,28,29]. However, the ideal site for anatomical tunnel localization remains unclear. While performing MPFL reconstruction, we used palpation to determine the FT location in our clinical practice in the past, but in recent years we have been using intraoperative fluoroscopy while referring to Schottle’s point. In our study, we found that the FT location was in the anatomical position in 22 (64.7%) patients, while the remaining 12 (35.3%) patients’ tunnel location was in the nonanatomical position. By palpation, Hopper et al. reported that 71.7% and Pandey et al. reported 63.3% of the FTs were in an acceptable position in their case series, while McCarthy et al. reported only 36% successful FT placement [8,18,22]. Koenen et al. compared the success of palpation with the use of intraoperative fluoroscopy. They recommended the use of intraoperative fluoroscopy in daily surgical practice, as the use of fluoroscopy improves accuracy in FT positioning [30]. We also think that tunnels should be opened as close to their anatomical locations as possible to provide postoperative physiological patellofemoral kinematics and pressure, and we agree with Koenen et al.’s recommendation for the routine use of intraoperative fluoroscopy to increase the accuracy of FT placement.

The present study had several limitations. It was a retrospective study in which data were collected prospectively. Other limitations of the study were the small number of patients, the lack of preoperative functional scores, the inability to measure patellar tunnel locations, and the fact that it is not clear how much deviation from Schottle’s point is acceptable when classifying FTs as anatomical or nonanatomical.

## 5. Conclusion

It is still unclear how to obtain the best results in patients with patellofemoral instability. In the current study, although there was no difference between the functional and clinical results according to the FT location of the patients who underwent the double patellar tunnel technique, a statistically significant difference was found in the VAS pain score. Pain in patients with a nonanatomical tunnel location may be due to changes in PFJ kinematics. In order to understand the effect of FT placement on the PFJ, long-term, prospective, randomized clinical studies with larger samples are needed.

## Figures and Tables

**Table 1:** Descriptive data for the anatomical/nonanatomical femoral tunnel groups.

Femoral tunnel	Anatomic (n = 22)		Nonanatomic (n = 12)		Statistical analysis^*^
					Probability
Variable	Mean ± SD	Median [min–max]	Mean ± SD	Median [min–max]	

Age (years)	22.13 ± 7.63	19.5 [16.0–37.0]	24.46 ± 10.71	19 [15.0–41.0]	z = 0.000 p = 1.000
Tunnel distance (mm)	2.14 ± 0.55	2.1 [1.2–2.8]	10.87 ± 4.94	9.8 [5.0–22.8]	t = –7.657 p = 0.000
	**n**	**%**	**n**	**%**	
**Operated side**					
Right	6	27.3	7	58.3	p = 0.139
Left	16	72.7	5	41.7	
**Sex**					
Male	8	36.4	3	25	p = 0.631
Female	14	63.6	9	75	
**Complication**					
No	22	100	10	83.3	p = 0.118
Yes	-	-	2	16.7	

**Table 2:** Comparison of VAS pain scores between the anatomic/nonanatomical tunnel groups.

Femoral tunnel	Anatomic (n = 22)		Nonanatomic (n = 12)		Statistical analysis^*^
					Probability
VAS	Mean ± SD	Median [min–max]	Mean ± SD	Median [min–max]	
Preoperative	5.00 ± 3.51	4 [1.0–10.0]	6.62 ± 2.26	7 [3.0–10.0]	z = –1.292
					p = 0.212
Postoperative	0.50 ± 0.76	0 [0.0–2.0]	2.08 ± 1.75	2 [0.0–6.0]	z = −2.443
					**p = 0.015**
**Statistical analysis**	z = −2.527		z = −3.189		
**Probability**	**p = 0.012**		**p = 0.001**		

**Table 3:** Comparison of functional scores by anatomical/nonanatomical tunnel groups.

Femoral tunnel	Anatomic (n = 22)		Nonanatomic (n = 12)		Statistical analysis* Probability
Variable	Mean ± SD	Median [min–max]	Mean ± SD	Median [min–max]	
Kujala	78.88 ± 20.56	83.5 [33.0–96.0]	74.62 ± 18.07	82 [39.0–100.0]	z = –0.653 p = 0.514
Tampa kinesiophobia	43.00 ± 7.01	41 [32.0–53.0]	43.15 ± 7.13	42 [32.0–58.0]	z = –0.254 p = 0.799
